# Effects of tumour acidification with glucose+MIBG on the spontaneous metastatic potential of two murine cell lines

**DOI:** 10.1038/sj.bjc.6601766

**Published:** 2004-04-13

**Authors:** T Kalliomäki, R P Hill

**Affiliations:** 1 Experimental Therapeutics Division, Ontario Cancer Institute/Princess Margaret Hospital, Toronto, Ontario, Canada M5G 2M9; 2 Department of Medical Biophysics; 3 Department of Radiation Oncology, University of Toronto, 610 University Avenue, Toronto, Ontario, Canada M5G 2M9

**Keywords:** acidic pH, MIBG, hypoxia, metastasis, murine tumours

## Abstract

In addition to hypoxia, acidic extracellular pH (pH_e_) is recognised as one of the microenvironmental characteristics of solid tumours. A number of studies have examined ways to increase tumour acidity in order to improve tumour-specific targeting of certain drugs and the effectiveness of hyperthermia. However, previous data have shown that exposure of murine tumour cells to acid conditions in culture can enhance their metastatic potential when injected subsequently into mice, raising the concern that deliberate tumour acidification might increase the probability of metastasis. In this study, we examined the effects of *in vivo* tumour acidification and hypoxia on the spontaneous metastatic potential of the murine KHT-C fibrosarcoma and B16F1 melanoma cell lines. A tumour-specific increase in extracellular acidity, demonstrated by measurements with pH electrodes, was achieved by daily intraperitoneal injections of meta-iodo-benzylguanidine (MIBG) and/or glucose. This method of tumour acidification during tumour growth did not significantly enhance the spontaneous metastatic potential of the two murine cell lines.

Tumour acidity is probably caused by excessive production of lactic and carbonic acids and their insufficient clearance by abnormal tumour vasculature (Newell *et al*, 1993; Stubbs *et al*, 2000; Griffiths *et al*, 2001). Several studies have examined ways to further reduce extracellular pH (pH_e_) in order to improve the therapeutic index of pH responsive cancer therapies (Wike-Hooley *et al*, 1984; Thistlethwaite *et al*, 1987; Tannock and Rotin, 1989; Jahde *et al*, 1992; Kuin *et al*, 1994). Acidification of the tumour extracellular space with glucose alone or in combination with meta-iodo-benzylguanidine (MIBG) provides a means for preferential intracellular accumulation and enhanced cytotoxicity of weakly acidic chemotherapeutic drugs, such as chlorambucil (Kuin *et al*, 1999; Kozin *et al*, 2001) and increases tumour cell sensitivity to hyperthermia (Ohtsubo *et al*, 2001). Acidification of human colonic xenografts has also been shown to enhance their sensitivity to aminolevulinic acid-mediated photodynamic therapy (Piot *et al*, 2001).

Acidic pH has also been implicated in progression of cultured human melanoma cells, which were found to have increased invasiveness through Matrigel following acidic culture conditions (Martinez-Zaguilan *et al*, 1996). A similar observation was made for metastatic B16 mouse melanoma cells where an increase in invasiveness was accredited to an acidosis-induced increase in expression of a gelatinase thought to be gelatinase B (matrix metalloproteinase 9). The relative gelatinase activity was further shown to be in good agreement with the different metastatic potentials of three different B16 subclones (Kato *et al*, 1992). In separate studies, the ability of murine B16F1 melanoma and KHT fibrosarcoma cells to form lung metastases after intravenous injection was shown to increase significantly after exposure to acidic conditions in culture (Schlappack *et al*, 1991; Jang and Hill, 1997). In humans, elevated tumour lactate concentrations, which generally correlate with reduced pH_e_, have been found to predict the likelihood of metastatic disease in head and neck and cervical carcinomas (Parkins *et al*, 1997; Walenta *et al*, 2000; Brizel *et al*, 2001). In addition to upregulated expression of invasion-enhancing proteins such as gelatinases, this increase in metastases may be related to acidosis-induced elevation of interleukin-8 (IL-8) and/or vascular endothelial growth factor (VEGF), factors known for their angiogenic potential (Shi *et al*, 1999; Xu and Fidler, 2000; Fukumura *et al*, 2001; Shi *et al*, 2001).

High lactate concentrations and low pO_2_ levels in tumours have been shown to correlate with increased likelihood of metastatic disease (Walenta *et al*, 2000; Brizel *et al*, 2001; Cairns *et al*, 2001; Hill *et al*, 2001). However, low pO_2_ levels and acidic pH_e_ are also known to coexist in solid tumours and thus it has been difficult to separate the effects of these two factors on metastatic potential *in vivo* (Helmlinger *et al*, 1997; Rofstad, 2000; Hill *et al*, 2001). To clarify further the relationship between acidic pH and metastatic tumour progression, we injected MIBG in combination with glucose into tumour-bearing mice; this treatment results in selective tumour acidification by inhibiting mitochondrial respiration at complex I of the electron transport chain and by stimulation of lactic acid production through anaerobic glycolysis (Loesberg *et al*, 1990; Jahde *et al*, 1992; Kuin *et al*, 1994, 1999). We examined the relative contribution of oxygenation and acidity to the spontaneous metastatic potential of the murine KHT-C fibrosarcoma and B16F1 melanoma cells by treating tumour-bearing mice daily with glucose+MIBG alone or in combination with a strategy to simulate acute hypoxia (Cairns *et al*, 2001).

## MATERIALS AND METHODS

### Animals and tumours

The experiments were carried out with two previously described murine cell lines: KHT-C fibrosarcoma and B16F1 melanoma, which were maintained using an alternating *in vivo*–*in vitro* growth protocol (Bristow *et al*, 1990). Cells were propagated *in vitro* in *α*-MEM media supplemented with 10% foetal calf serum and antibiotics. For *in vivo* experiments 2–5 × 10^5^ cells were injected intramuscularly into the gastrocnemius muscle of the left hind leg of 8–12-week old C3H-HeJ male (KHT-C) and C57Bl/CRL female (B16F1) mice to produce tumours as described previously (Cairns *et al*, 2001). Tumour growth was monitored daily by leg diameter measurements, which were converted into tumour weight by a previously made standard curve. Animals were maintained in the Animal Resource Centre of the Ontario Cancer Institute/Princess Margaret Hospital and all procedures on the animals had the ethical approval of the Animal Care Committee in compliance with the guidelines of the Canadian Council on Animal Care. These guidelines meet the standards required by the UKCCCR (Workman *et al*, 1998).

### pH_e_ and pO_2_ measurements

Prior to and at 1 and 3 h following glucose±MIBG injections, mice with tumours weighing ∼0.4–0.5 g (∼9.0–10.0 mm) were restrained in a jig. Following a two-point calibration (Fisher Scientific standardising buffers 4.01 and 7.00; slope min 80%), a 21G needle electrode (type MI-418, Microelectrodes Inc., Bedford, NH, USA connected to ORION pH meter, model 230A, Boston MA, USA) was inserted into the tumour through a premade 21G needle puncture in the skin and withdrawn in a stepwise manner to record pH values at different depths. A reference electrode (type MI-402, Microelectrodes Inc., Bedford, NH, USA) was placed subcutaneously in the same leg. Any drift caused by protein contamination of the needle probe was assessed by placing both of the probes back into pH 7.00 standardising buffer after the measurement of each tumour and by recording the difference that had occurred during the measurement. All of the pH values presented are corrected for their individual drifts, which on average was +0.03 pH units following approximately 10 min measurement interval. This drift increased significantly with increasing measurement time, thus preventing accurate measurements of long-term pH_e_ changes in the tumours. The probes were rinsed with distilled water and recalibrated with pH 7.00 buffer following measurements in every mouse.

Measurements of tissue oxygenation were made using the fibreoptic-based OxyLite system (Oxford Optronix, Oxford, UK) as described previously (Cairns *et al*, 2001) in a restraint set-up identical to that used for pH_e_ measurements. The probe was placed initially in a region of the tumour with a pO_2_ value around 5–10 mmHg and then left in place for a number of hours. Data recording was initiated within 30 min of injection with glucose+MIBG. To obviate the possibility of metastatic contribution by dislodged tumour cells, no needle electrode or pO_2_ measurements were made in the tumours of mice used to generate the spontaneous metastasis data. All measurements were carried out without general anaesthetics.

### Tumour treatment protocol for metastasis experiments

Treatment to induce tumour acidification was started when the diameter of the tumour-bearing leg reached 7.5 mm (0.23 **g**; about 5–7 days after tumour inoculation). Earlier initiation of treatment resulted in a significant inhibition of tumour growth. Mice in the treatment group were injected with 30 mg kg^−1^ MIBG (a gift from Dr Dewhirst, Durham, NC, USA) in phosphate-buffered saline (PBS) intraperitoneally (i.p.), immediately followed by 3 g kg^−1^ glucose (i.p.) in distilled water. Owing to different tolerance levels, the drug was administered in different schedules to the two different mouse strains. The C3H-HeJ males with KHT-C tumours received glucose+MIBG on every other day and on the intervening day only glucose was administered at a dose of 3 g kg^−1^. The C57Bl/CRL females with B16F1 tumours received glucose+MIBG on two consecutive days followed by a day on which only glucose was administered at a dose of 3 g kg^−1^. Control animals received an equal number of daily PBS injections. This treatment schedule was repeated until a predetermined tumour weight was reached. In two experiments, a separate group of KHT-C tumour-bearing animals were treated with glucose alone, receiving single daily doses of glucose at 3 g kg^−1^.

In experiments identified as ‘combination treatments’, the above injections were given together with daily treatments to simulate acute hypoxia. This consisted of 12 alternating breathing cycles of 10 min 7% O_2_, balance N_2_ and 10 min air for the treatment animals and of 240 min air breathing for the control animals. In the treatment animals, this resulted in pO_2_ fluctuations between approximately 40 and 10 mmHg in normal muscle and between <10 and near 0 mmHg in the tumour. The acute hypoxia treatment was started on the day following tumour cell inoculation as was done previously (Cairns *et al*, 2001). During the days when animals received the combination treatment, control mice were injected first (with PBS) followed by the treatment group (with glucose±MIBG). Immediately following the i.p. injections, animals were exposed to either the control or acute treatment conditions of the gassing programme.

Mice with KHT-C tumours were killed when the tumour weight reached 0.45±0.05 **g** (9.0±0.5 mm; 4–5 days of tumour-acidifying treatment). Owing to their higher drug tolerance levels and lower metastatic efficiency, mice with B16F1 tumours were killed when the tumour weight reached 1.7±0.17 **g** (15.0±0.5 mm) or a maximum of 6 days of tumour-acidifying treatment, whichever end point was reached first. The lungs were then removed for histological assessment of micrometastases where four individual 5 *μ*m sections, separated by at least 200 *μ*m, were obtained from each lung and examined as described previously (Cairns *et al*, 2001). Experiments examining metastasis with acute hypoxia alone or in combination with glucose+MIBG injections in the KHT-C model were repeated four times with 5–12 mice per repeat group with total number in each group ranging from 28 to 33. Glucose with or without acute hypoxia experiments in the KHT-C model was repeated twice with 7–13 animals per repeat group and 17–23 animals in total. A single experiment with 13–15 animals per group was carried out to examine metastasis in the B1F1 model.

### Tumour treatment protocol for nonmetastasis experiments

Additional mice were used to assess the effects of previously described tumour microenvironmental manipulations on tumour pH_e_. Generally, pretreatment measurements were obtained 1 day prior to or on the day of treatment. For measurements of tumour pH_e_ following acute hypoxia, one mouse was removed from the treatment chamber and tumour pH_e_ was measured during the first reoxygenation cycle of the hypoxia treatment. The next mouse was measured during the second reoxygenation cycle and this was repeated until all of the animals were measured during ∼160 min of acute hypoxia. When this treatment was combined with tumour-acidifying injections, mice were injected with one dose of glucose+MIBG prior to treatment with acute hypoxia and were measured as described for acute hypoxia treatment alone. Mice used to examine the kinetics of pH_e_ reduction received multiple injections in a schedule similar to that used with animals in the metastasis experiment.

### Statistical analysis

The Student's *t*-test with Bonferroni correction was applied to the normally distributed data sets (the pH_e_ values). The Kruskal–Wallis ranking test was used on the nonparametric data sets with three or more treatment groups with the limit of statistical significance set at *P*<0.05. A power analysis of the metastasis data was performed to determine its ability to detect a difference. As the data is not normally distributed, it was log-normalized and the pooled variance was calculated. Methods described by Bausell and Li (2002) were used to determine the minimum difference that could have been detected with a *P*-value of 0.05 and a power of 80%.

## RESULTS

### Effects of glucose±MIBG and acute hypoxia on tumour pH_e_

**Figure 1:**
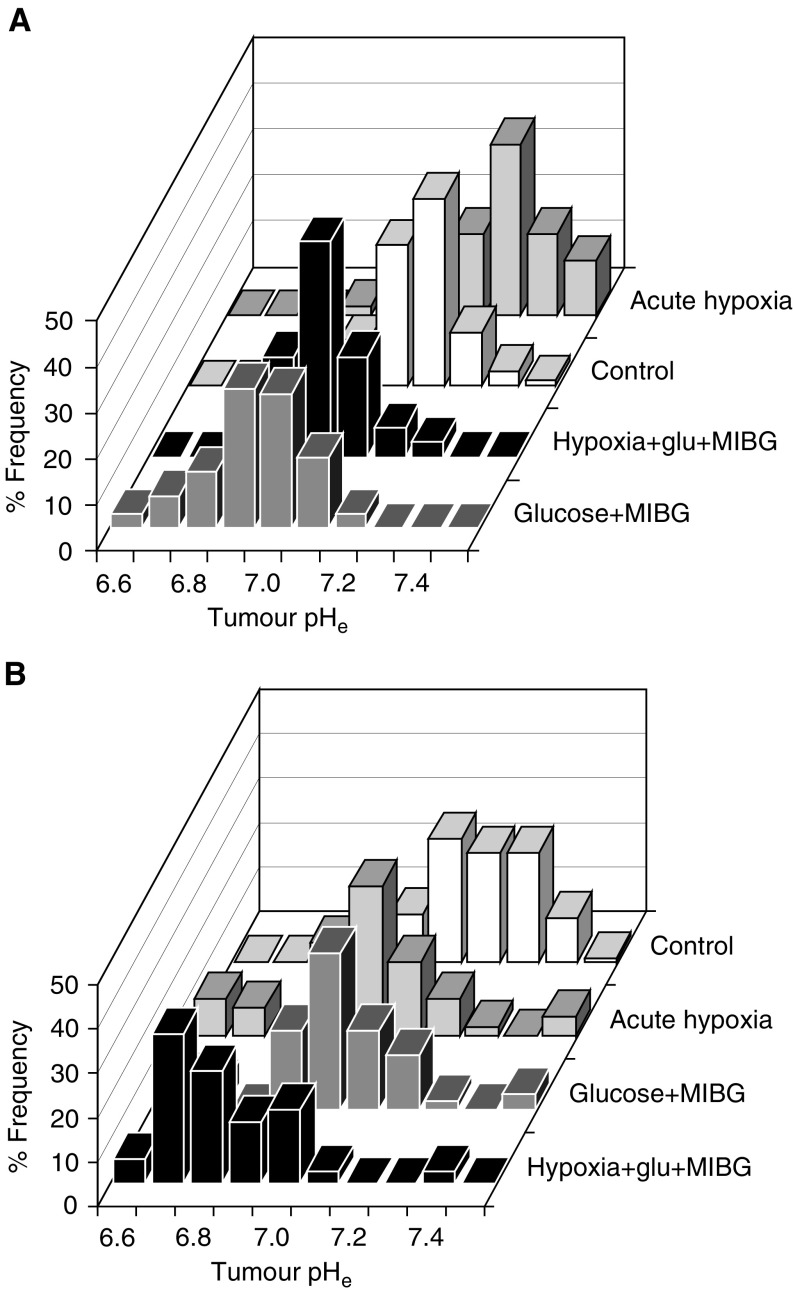
Histograms of pooled pH_e_ values before and after treatment in KHT-C and B16F1 tumours. (**A**) KHT-C: Control: pretreatment measurements from animals used for post-treatment measurements, obtained 1 day prior to or on the day of treatment. Mean pH_e_=7.13±0.017; *N*=25; *n*=179. Glucose+MIBG: Animals treated with 3 g kg^−1^ glucose+30 mg kg^−1^ MIBG were measured 1 h postinjections. Mean pH_e_=6.89±0.030; *N*=12; *n*=99. Acute hypoxia: mice were measured during reoxygenation cycles of 160 min of acute hypoxia treatment. Mean pH_e_=7.23+0.045, *N*=8; *n*=51. Acute hypoxia+glucose+MIBG: animals were injected with one dose of glucose+MIBG prior to acute hypoxia and were measured as described for acute hypoxia alone. Mean pH_e_=6.97±0.031; *N*=5; *n*=32. (**B**) B16 F1: animals were measured as described for the KHT-C tumours, unless specified otherwise. Control: all pretreatment measurements were made on the day of the first treatment. Mean pH_e_=7.14±0.022; *N*=19; *n*=152. Glucose+MIBG: measurements were carried out following 1–3 rounds of injections on days 7–9. Mean pH_e_=6.89±0.064; *N*=6; *n*=48. Acute hypoxia: mean pH_e_=7.01±0.063; *N*=4; *n*=33. Acute hypoxia+glucose+MIBG: mean pH_e_=6.79±0.042; *N*=4; *n*=36. (mean±1 s.e.m.; *N*=the number of animals used; *n*=pooled number of pH readings obtained).

Pooled pH_e_ values measured before and approximately 1 h after treatment demonstrate the degree of acidification achieved with various treatments in the KHT-C (Figure 1A

) and in the B16F1 (Figure 1B) tumours. Maximal acidification of the KHT-C tumours (mean change in pH_e_: 0.24 U; *P*=1.0 × 10^−8^) was observed following glucose+MIBG injections. This treatment also significantly acidified the B16F1 tumours (mean change in pH_e_: 0.25 U; *P*=6.6 × 10^−5^), as did acute hypoxia (mean change in pH_e_: 0.13 U; *P*=0.027), but the B16 F1 tumours were found to become most acidic when these two treatment modalities were combined (mean change in pH_e_: 0.35 U; *P*=8.2 × 10^−7^). In B16F1 tumours, the acute hypoxia+glucose+MIBG-treated group is significantly different from the glucose+MIBG-treated group (*P*=3.35 × 10^−9^). In the KHT-C tumours, addition of acute hypoxia to glucose+MIBG injections increased mean tumour pH_e_ as compared to the injections alone (mean difference: 0.08 U). Acute hypoxia alone also increased KHT-C tumour pH_e_ (mean change in pH_e_: 0.10 U; *P*=0.016), but the biological significance of this increase remains undetermined. In accordance with previous reports (Jahde *et al*, 1992; Zhou *et al*, 2000), glucose+MIBG injections had no significant effect on normal tissue (untreated muscle pH_e_: 7.00±0.074 *vs* 7.07±0.046 s.e.m. 1 h postinjections).

**Figure 2:**
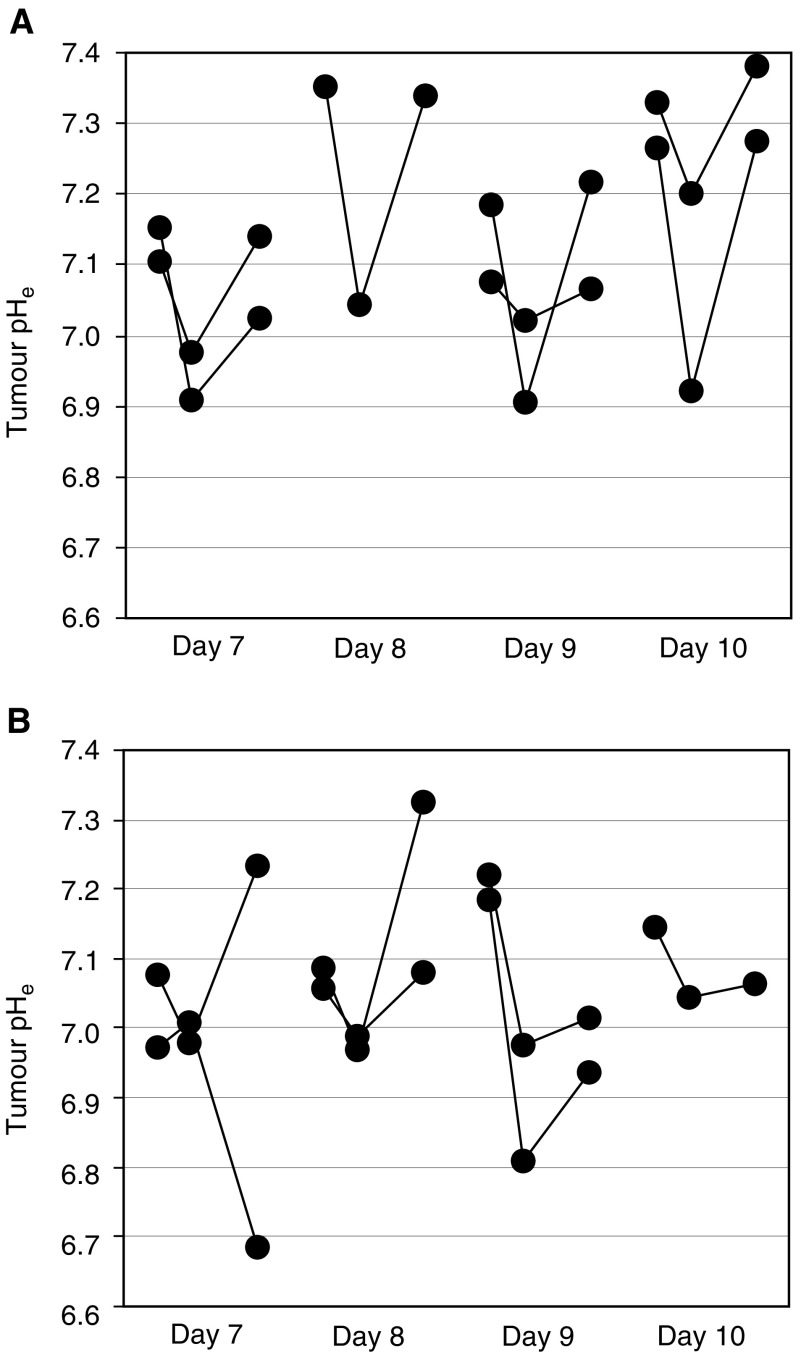
Kinetics of pH_e_ reduction following tumour-acidification treatments on four consecutive days in KHT-C and B16 F1 tumours. The distribution of pH_e_ was measured daily in total of seven different tumour-bearing animals. One animal on day 8 in panel A was omitted due to a missed time point and another animal on day 10 in panel B died. The three connected circles for each animal represent pre-, 1 h post- and 3 h postinjection time points. Maximal reduction in mean pH_e_ of both tumour models was generally observed at the first time point, 1 h post-treatment and pH_e_ returned near the initial value by 3 h post-treatment. (**A**) KHT-C (*N*=7): pretreatment measurements were made on each day of the treatment during days 7–10. Injections consisted of 3 g kg^−1^ glucose+30 mg kg^−1^ MIBG on days 7 and 9 and of 3 g kg^−1^ glucose on days 8 and 10. (**B**) B16F1 (*N*=7): all pretreatment measurements were obtained on day 7. The animals were treated with 3 g kg^−1^ glucose+30 mg kg^−1^ MIBG on days 7–9 and with 3 g kg^−1^ glucose alone on day 10.

Examples of individual tumour responses during the first 4 days of tumour-acidification treatment with glucose+MIBG are shown in Figure 2

. In most tumours, measurements of mean pH_e_ values in individual mice showed maximal acidification within 1 h after injections and return of these values near to their pretreatment values within 3 h of injection. The mean degree of KHT-C tumour acidification following glucose+MIBG injections ranged from 0.06 to 0.28 pH units. The mean degree and duration of tumour acidification achieved with less toxic glucose on days 8 and 10 for KHT-C (Figure 2A) appears equivalent to those achieved with glucose+MIBG on days 7 and 9, which would suggest the use of glucose alone for tumour acidification to limit the observed toxicity. However, when used without intervening MIBG injections, a dose of up to 6 g kg^−1^ glucose resulted in only a 0.1 U reduction in tumour pH_e_, which returned back to normal within 1 h (data not shown). This moderate tumour sensitivity to glucose suggests that the greater mean degree of tumour acidification (0.26 pH units) achieved with glucose alone in Figure 2A, is likely due to inflated pretreatment measurements, which on the days following glucose+MIBG injections, are 0.19 pH units above the mean untreated pH_e_ levels of 7.12. This apparent overcompensation of tumour pH_e_ in response to glucose+MIBG within 24 h of injections was taken into account in generating data for panel B, Figure 2, by making all pretreatment measurements prior to any injections, on day 7. In B16F1 tumours, glucose+MIBG injections resulted in reductions ranging from 0.10 to 0.38 pH units. Thus, although the mean pH_e_ in every tumour decreased after each daily treatment, heterogeneity in the responses of individual mice was evident.

### Effects of tumour acidification on pO_2_

**Figure 3:**
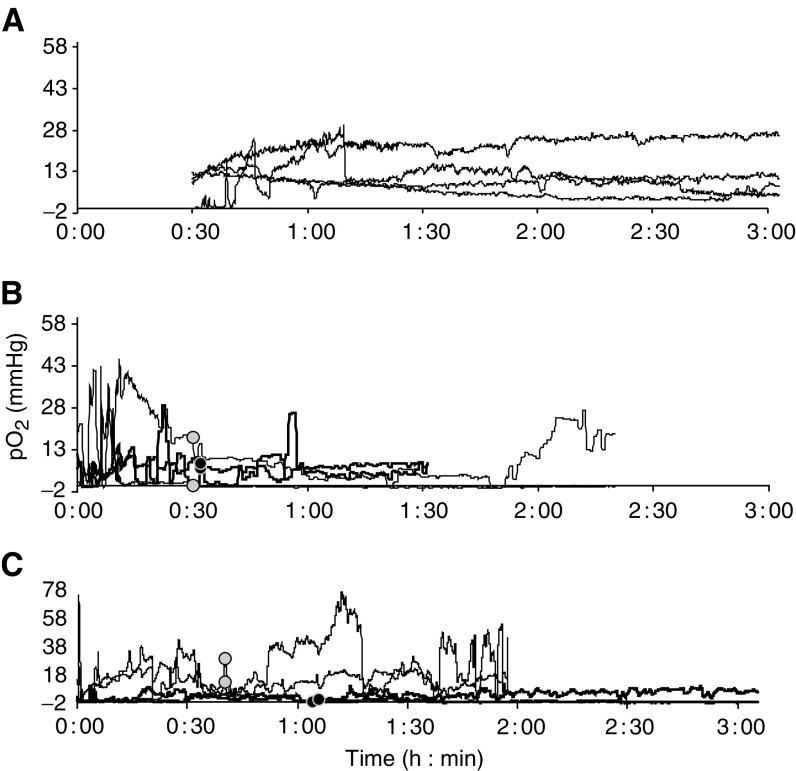
Oxygen tension measurements in KHT-C and B16F1 tumours treated with 30 mg kg^−1^ MIBG+3 g kg^−1^ glucose. Most tumours (seven out of eight) were concurrently monitored with two OxyLite probes. No consistent changes in pO_2_ levels in response to the injections were observed. (**A**) KHT-C tumours (*N*=4); measurements were started within 30 min of injections and continued up to 3 h after treatment. (**B**) KHT-C tumours (*N*=2); measurements were started prior to injections, marked by • and 
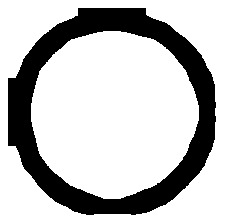
, and continued for 1–2 h after treatment. (**C**) B16F1 tumours (*N*=2); measurements were started prior to injections, marked by • and 
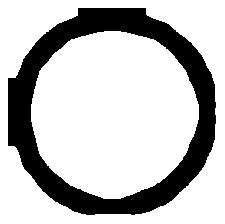
, and continued up to 2 h after treatment.

MIBG is a known inhibitor of mitochondrial respiration (Smets *et al*, 1988; Biaglow *et al*, 1998; Loesberg *et al*, 1990) and a hyperglycaemia-induced shift to glycolysis has been shown to alter cellular oxygen consumption (Nadal-Desbarats *et al*, 2002). Therefore, we examined whether the glucose+MIBG treatment could result in an increase in tumour pO_2_ as reported by others (Burd *et al*, 2001, 2003). The pO_2_ levels were measured in six KHT-C and two B16F1 tumours with two probes/tumour in all but one case. Recording of data was initiated within 30 min of glucose+MIBG injections in four KHT-C tumours (Figure 3A

) or prior to injections in two KHT-C (Figure 3B) and two B16F1 tumours (Figure 3C). As reported recently by Brurberg *et al* (2003), it can take up to 15 min for measurements with an OxyLite probe to stabilize, hence the early measurements shown in this figure should be viewed with caution. No consistent changes in tumour pO_2_ levels in response to tumour acidification were observed.

### Effects of tumour acidification and acute hypoxia on metastases

Results of microscopic lung metastasis data from four repeat experiments are shown individually in Table 1

**Table 1:** Median number of microscopic lung metastases in individual experimental repeats

	**Air**		**Hypoxia**	
	**PBS**	**Glu+MIBG**	**PBS**	**Glu+MIBG**
Repeat 1	5 (6)	24 (6)	17 (5)	14 (6)
Repeat 2	6 (5)	3 (5)	13 (6)	35 (6)
Repeat 3	27 (10)	20 (10)	35 (9)	14 (9)
Repeat 4	26 (12)	29 (12)	18 (8)	35 (8)
				
Median (pasted)	17	20	18	24
First/third quartiles	8/43	17/50	6/38	11/48

**Figure 4:**
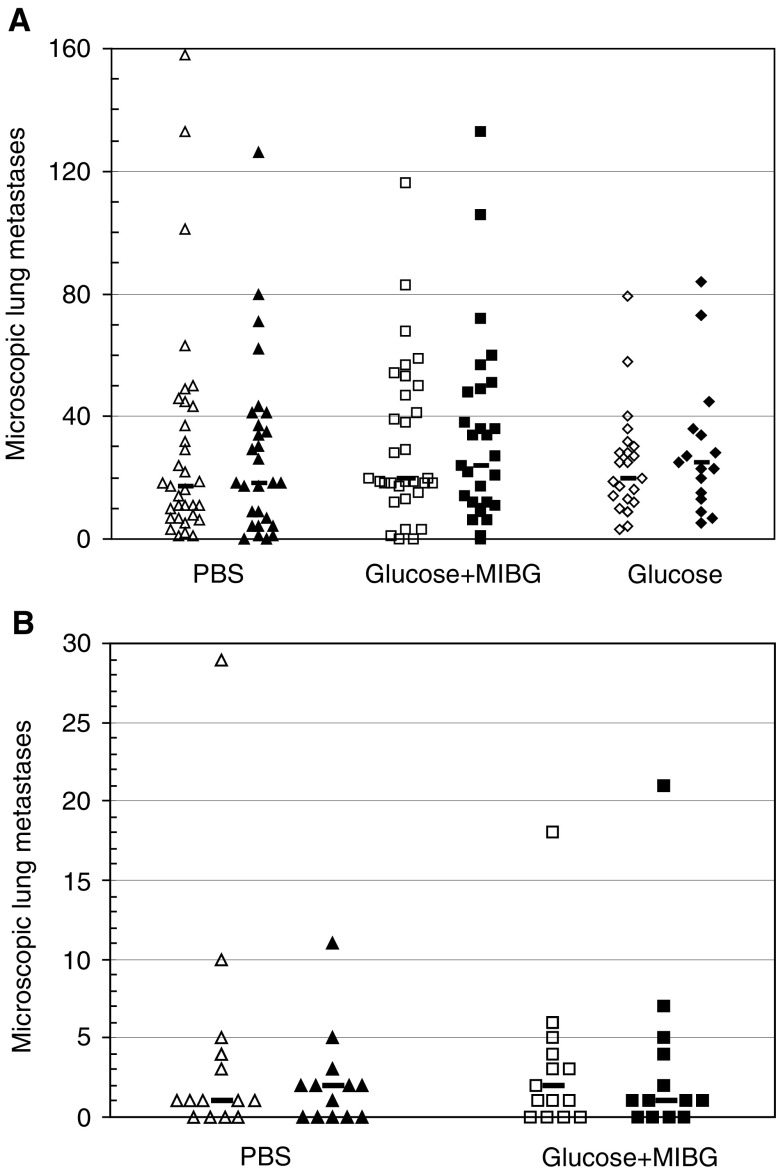
Microscopic lung metastases detected in KHT-C or B16F1 tumour-bearing animals treated with glucose+MIBG (□), glucose (⋄) or control injections (PBS, ▵) in combination with acute hypoxia or control air gassing conditions, indicated by solid and open symbols, respectively. (**A**) KHT-C: pooled medians from 2–4 repeat experiments (R): Air, PBS=17 (4R, *N*=33); Air, glucose+MIBG=20 (4R, *N*=33); Air, glucose=20 (2R, *N*=23); Hypoxia, PBS=18 (4R, *N*=28); Hypoxia, glucose+MIBG=24 (4R, *N*=29); Hypoxia, glucose=25 (2R, *N*=17). (**B**) B16F1: medians from one set of experiments: Air, PBS=1 (*N*=15); Air, glucose+MIBG=2 (*N*=15), Hypoxia, PBS=2 (*N*=14); Hypoxia, glucose+MIBG=1 (*N*=13).

Hypoxia gassing alone showed an increased number of lung metastases in three of four experiments, although no individual repeat showed statistical significance. Tumour acidification injections with glucose+MIBG resulted in a nonsignificant increase in the number of metastases in two of four experimental repeats both under air control and hypoxic gassing conditions. Number of experimental animals per group is indicated in parenthesis.

and the combined data is shown in Figure 4

. Tumour acidification with glucose+MIBG did not significantly increase the spontaneous metastatic potential of either cell line (KHT-C pooled median: control=17, treatment=20; B16F1 median: control=1, treatment=2). Although the degree of KHT-C tumour acidification achieved with daily glucose injections was less than that achieved glucose+MIBG, we also examined the effects of daily 3 g kg^−1^ glucose injections on metastatic efficiency (mean resultant pH_e_=7.06±0.017; *N*=5; *n*=34). The number of micrometastases in this group did not differ from the groups treated with PBS or glucose+MIBG (KHT-C pooled median: control=17, treatment=20). Treatment of the KHT-C tumours with acute hypoxia alone led to a nonsignificant increase in microscopic metastases in three of four experimental repeats (Table 1). None of the treatments had an effect on the metastatic potential of the B16F1 tumours.

## DISCUSSION

Current evidence suggests that the tumour microenvironmental characteristic, acidic pH_e_, can be exploited and enhanced to improve the efficacy of hyperthermia, certain forms of chemotherapy and photodynamic therapy (Wike-Hooley *et al*, 1984; Tannock and Rotin, 1989; Jahde *et al*, 1992; Kuin *et al*, 1994, 1999; Kozin *et al*, 2001; Ohtsubo *et al*, 2001; Piot *et al*, 2001). However, acidic pH and lactate have also been associated with enhanced metastatic tumour progression both in experimental models (Schlappack *et al*, 1991; Jang and Hill, 1997) and clinically (Parkins *et al*, 1997; Walenta *et al*, 2000; Brizel *et al*, 2001). We wanted to determine whether the glucose+MIBG-based tumour-acidifying treatment, used to improve efficacy of cancer therapies, would affect the spontaneous metastatic potential of two murine tumour cell lines, KHT-C and B16F1, previously shown to increase their experimental metastatic potential following acidic culture conditions (Schlappack *et al*, 1991; Jang and Hill, 1997).

Significant acidification of the two tumour types following glucose+MIBG injections was achieved. The mean reduction was 0.24 pH units for KHT-C and 0.25 pH units for B16F1 tumours. Although these reductions are smaller and subsequently return back to original levels faster than has been reported previously following glucose+MIBG treatment in mouse models (Jahde *et al*, 1992; Kuin *et al*, 1994), they are very similar to those achieved with glucose treatment alone in cancer patients without local anaesthesia where the mean reduction ranged from 0.16 to 0.19 pH units (Leeper *et al*, 1998). The difference between studies conducted in mice is likely a combination of the use of different mouse and tumour cell lines, different dose and route of glucose administration and a lower dose of MIBG, which was limited by toxicity. In the B16F1 tumours, which were grown in female mice, this may also be due partly to the sex of the animals since Kuin *et al* (1994) have previously reported that the degree of RIF-1 tumour acidification was less in female mice than in identically treated male mice.

Despite previously demonstrated cyclical reduction in tumour pO_2_ during acute hypoxia and evidence for enhanced glycolysis rates following hypoxic culture conditions (Skoyum *et al*, 1997; Cairns *et al*, 2001), acute hypoxia resulted in a small reduction in pH_e_ only in the B16F1 model. The mean change from the untreated control values was +0.10 pH units for the KHT-C (range: from −0.15 to +0.41 pH units) and −0.13 pH units for the B16F1 tumours (range: from −0.26 to +0.14 pH units; Figure 1). The dissimilarity of these two different tumour cell lines in their responses to acute hypoxia may represent an extension of previous reports outlined in the thorough review by Wike-Hooley *et al* (1984) where the mean pH values of untreated mouse tumours were reported to vary from a mean of 6.74 (range 6.65–6.93) in a hepatoma to a mean of 7.1 (range 6.8–7.4) in a mammary adenocarcinoma.

It was also anticipated that hypoxia-induced oxygen deficiency together with the mitochondrial respiration inhibitor, MIBG, might further shift energy production from aerobic to anaerobic glycolysis with subsequent increase in acid production and further decrease in tumour pH_e_. Despite the variability in individual mouse responses to acute hypoxia treatment alone, this appears to hold true for the B16F1 tumours as the reduction of 0.35 U in mean tumour pH_e_ following the combination treatment is close to the added mean values of 0.13 and 0.25 pH units from acute hypoxia and glucose+MIBG treatments, respectively. The degree of acidification observed with the combination treatment in the KHT-C model (mean change in pH_e_: 0.16 U) was less than that seen with glucose+MIBG alone (mean change in pH_e_: 0.24 U), which is again consistent with the increase in pH_e_ induced by acute hypoxia (0.10 pH units). However, the reason for the increase in pH_e_ due to exposure to acute hypoxia remains unclear. Tumour cell metabolism has been found to be limited primarily by substrate availability, such as glucose for anaerobic glycolysis, rather than the metabolic demands by tumour cells (Kallinowski *et al*, 1988; Helmlinger *et al*, 2002). Therefore, it seems possible that the combination treatment of the KHT-C model caused such a severe depletion of energy stores that little additional acidification from the pre-existing levels was attainable. This alternative is supported by studies by Rotin *et al* (1986) in which culturing cells under acidic and hypoxic conditions led to a decrease in cellular ATP levels and in the rates of glucose consumption and lactate production. These data are also consistent with the observation where the combination treatment slightly inhibited (<1 day) the development of KHT-C tumours although it had no effect on the growth rate of B16F1 tumours (data not shown).

Previous studies have reported that MIBG can reduce oxygen consumption by tumour cells (Smets *et al*, 1988; Loesberg *et al*, 1990; Biaglow *et al*, 1998;), therefore we examined whether our tumour-acidifying protocol had secondary effects on tumour oxygenation. The KHT-C fibrosarcoma used in this study has previously been shown to be hypoxic with HP_5_ values (tumour pO_2_ values <5 mmHg) ranging from 25–100% in individual tumours (median 68%); similar values have been reported in clinical soft tissue sarcomas with HP_5_ values ranging from 0–76% (Brizel *et al*, 1994; Nordsmark *et al*, 1996; De Jaeger *et al*, 2001). We observed no consistent change in tumour pO_2_ levels following glucose+MIBG injections in either tumour model (Figure 3). If the increase in pO_2_ levels were immediate, it is possible that the increase would have been missed in our initial study (panel A), as it took approximately 30 min to start pO_2_ recording after the injections. However, a group of pO_2_ measurements (panels B and C) were made in a set-up that allowed glucose+MIBG injections to be administered without removing the OxyLite probes and again no consistent changes in pO_2_ levels were observed. A possible explanation for the lack of a detectable change is offered by Kallinowski *et al* (1989), who postulated that a decrease in oxygen consumption by tumour cells may be concealed by an up to 70-fold elevation in oxygen consumption by immune cells. Approximately 40% of cells from dissociated KHT-C tumours have been reported to be immune cells, primarily macrophages (Siemann *et al*, 1981). Alternatively, this observation may be due to the absence of anaesthetics in the current study, use of which has been reported to influence tissue pH_e_ and pO_2_ (Buelke-Sam *et al*, 1978; Kerger *et al*, 1997). These data suggest that the glucose+MIBG-mediated increase in tumour oxygenation and subsequent increase in radiosensitivity reported by Burd *et al* (2001, 2003) may be applicable only to certain tumours.

The absence of a change in the spontaneous metastatic potential in response to *in vivo* tumour acidification (Figure 4) was unexpected and may be due to inherent differences between the *in vitro* and *in vivo* systems. With the KHT-C tumours, the degree of acidification achieved with glucose+MIBG was significant, but was applied only during the second half of total tumour development time for a total of approximately 4 days. While this is consistent with any likely clinical application of such a treatment, it is a considerably shorter time and reduced treatment frequency than that used with animals treated with acute hypoxia. MIBG also had significant growth inhibitory effects on the KHT-C tumours if the injections were started before a palpable tumour was present. Although cell lines are generally highly capable of maintaining alkaline intracellular pH (pH_i_) even when the pH_e_ is as low as 6.2 (Lee and Tannock, 1998; Kozin *et al*, 2001), there is evidence that suggests that MIBG acidifies tumour cell pH_i_ via an unknown mechanism (Biaglow *et al*, 1998; Kuin *et al*, 1999; Zhou *et al*, 2000). It is therefore possible that MIBG-induced cytotoxic effects may have counteracted the potential metastasis promoting effects of tumour acidification. In an attempt to examine less toxic methods to achieve tumour acidification and to increase the number of days when the treatment was implementable, we tested carbogen (95% O_2_/5% CO_2_) breathing, which has been reported to result in significant tumour-specific acidification without growth inhibition (Stubbs *et al*, 1998). However, we saw no change in mean pH_e_ of the KHT-C tumours following 2 h of carbogen breathing (data not shown). To rule out the potential of MIBG-induced increases in tumour oxygenation and subsequent possibility of inhibition of metastasis formation, the tumour-acidifying treatment was also combined with acute hypoxia. The median number of lung metastases in this combination treatment group did not differ from the medians of the groups receiving either treatment alone. We believe that this addition of hypoxia to the tumour-acidifying regime should have been able to compensate any possible increases in tumour oxygenation and thus prevent masking of a positive effect of acidification on metastasis formation. We also examined the power of our data set to detect a difference. Based on this analysis, the data for MIBG+glucose has 80% power to detect a difference greater than 2.7-fold in metastasis formation. We conclude that if the pH_e_ changes induced by the treatment do cause an effect, it is unlikely to be larger than a 2.7-fold difference.

Owing to higher tolerance to MIBG, the B16F1 tumour model received tumour-acidifying injections longer and more frequently than the KHT-C model. The maximal degree of acidification was also higher in the B16F1 model (0.35 pH units *vs* 0.24 pH units for KHT-C). Despite this, no increase in metastases was observed contrary to our previous *in vitro* studies (Schlappack *et al*, 1991; Jang and Hill, 1997). The maximal mean reduction achieved *in vivo* remained significantly lower than the reduction of 0.7 pH units achieved *in vitro* (control pH 7.2, treatment pH 6.5). The acidic conditions were also maintained much longer *in vitro* (48 h, followed by a 24–48 h recovery period) than *in vivo* (<3 h). Thus, the acidic exposure *in vitro* was much more severe than could be achieved *in vivo*, which may explain the differences in the results obtained. This outcome may also be partially due to the low spontaneous metastatic efficiency of the B16F1 cell line. It is well known that growth site of the primary tumour can have a significant impact on the (metastatic) outcome of any given experiments and the predominant view supports an orthotopic rather than nonorthotopic site for maximal spontaneous metastatic effect (Manzotti *et al*, 1993; Blouw *et al*, 2003). It is possible that in our case, intradermally injected tumours might have given more metastases, however, such tumours tend to ulcerate at small sizes and we felt that they would be unsuitable for a study involving multiple needle insertions (personal observation, R Kuba).

In light of our previous data, which showed a significant increase in experimental metastatic potential by KHT-C and B16F1 cells following acidic culture conditions, the absence of an increase in spontaneous metastases underscores the complexity associated with the effect of the tumour microenvironment on malignant disease progression. Together with studies using noninvasive, *in vivo* pH quantifying imaging techniques, such as those described Gillies *et al* (2002), the current study, attaining a degree of tumour-acidification matching that achieved in the clinics, should provide valuable information about the potential risk–benefit ratio of tumour-acidification treatment in humans.
